# Heading Estimation for Pedestrian Dead Reckoning Based on Robust Adaptive Kalman Filtering

**DOI:** 10.3390/s18061970

**Published:** 2018-06-19

**Authors:** Dongjin Wu, Linyuan Xia, Jijun Geng

**Affiliations:** School of Geography and Planning, SunYat-Sen University, 135 # Xingangxi Road, Guangzhou 510275, China; gengjj@mail2.sysu.edu.cn

**Keywords:** heading estimation, robust adaptive Kalman filtering, pedestrian dead reckoning, MEMS sensors, smart phone

## Abstract

Pedestrian dead reckoning (PDR) using smart phone-embedded micro-electro-mechanical system (MEMS) sensors plays a key role in ubiquitous localization indoors and outdoors. However, as a relative localization method, it suffers from the problem of error accumulation which prevents it from long term independent running. Heading estimation error is one of the main location error sources, and therefore, in order to improve the location tracking performance of the PDR method in complex environments, an approach based on robust adaptive Kalman filtering (RAKF) for estimating accurate headings is proposed. In our approach, outputs from gyroscope, accelerometer, and magnetometer sensors are fused using the solution of Kalman filtering (KF) that the heading measurements derived from accelerations and magnetic field data are used to correct the states integrated from angular rates. In order to identify and control measurement outliers, a maximum likelihood-type estimator (M-estimator)-based model is used. Moreover, an adaptive factor is applied to resist the negative effects of state model disturbances. Extensive experiments under static and dynamic conditions were conducted in indoor environments. The experimental results demonstrate the proposed approach provides more accurate heading estimates and supports more robust and dynamic adaptive location tracking, compared with methods based on conventional KF.

## 1. Introduction

The expansion of location-based services (LBS) and applications has led to extensive interest in ubiquitous localization which may rely on widely used smart phones. The rich sensors embedded in smart phones support vary types of localization techniques, such as cellular localization, WiFi localization, vision-based location tracking, micro-electro-mechanical system (MEMS) sensors-based pedestrian dead reckoning (PDR), etc. However, a stand-alone technique cannot satisfy all positioning demands of LBS. For example, Global Navigation Satellite Systems (GNSS) cannot work in blocked regions, and WiFi localization systems (WLS) are limited by the coverage of WiFi signals, etc. Among these techniques, PDR is of great importance that it can flexibly link up different absolute positioning systems (such as GNSS, WLS, etc.) to achieve ubiquitous location provision for LBS. However, as a kind of relative localization method, it suffers from the problem of location error accumulation, and therefore cannot hold its performance continuously. Thus, it is essential to improve the tracking performance of the PDR method. Although external techniques, such as indoor graph matching [1,2,3], WiFi localization [3,4,5,6,7], visible light positioning [8] etc. have been considered to assist PDR, it is crucial to enhance its own performance in several aspects, such as speed estimation, heading determination and position calculation, the errors of which can propagate and can result in fast divergence of the localization performance. Therefore, this paper focuses on one aspect regarding heading estimation based on MEMS sensors.

Commonly, heading estimation based on MEMS sensors relies on the fusion of acceleration data from the accelerometer, magnetic field data from the magnetometer, and angular rates output by the gyroscope. It is known that accelerations and magnetic fields can be used to estimate absolute headings, whereas angular rates can be integrated iteratively to produce relative headings. Moreover, other combinations of the three sensors can also be applied for heading estimation. Because of the inconsistency of coordinate frames between a user and his/her device, different holding modes of the device require different solutions to derive the user’s headings. For example, when a smart phone is in the pocket, its attitude heading is dynamically changing, and thus it is hard to obtain the user’s heading through this smart phone. Account for this, some indirect methods [1,9] were proposed to determine headings by using the principle component analysis (PCA) algorithm for obtaining the largest variations of the horizontal plane of accelerations. Other holding modes, such as holding the smart phone in hand, holding against the ear always result constant misalignment of coordinate frames between the smart phone and the user, and the user’s heading is relatively easy to be determined by combining the smart phone’s attitude heading and a corresponding rotation matrix. Regarding the steady holding modes, the device attitude heading should be estimated first by fusing the aforementioned sensor data. The fusion is always by means of complementary filters (CFs) [10,11,12] and Kalman filters (KFs) [13,14,15,16,17].

CF is relatively easy to implement and has a low computational cost. However, the heading accuracy produced by CF is slightly lower than that by KF, and even worse in highly dynamic environments. For example, Wu et al. [10] proposed a kind of iteration-free fast complementary filter (FCF) for attitude estimation, and compared it with the linear Kalman filter (LKF) [13] using smoothly collected data. The experimental results show that the computational time cost of FCF decreases significantly, but the accuracy of FCF is a little lower than that of LKF. However, comparisons on dynamic data were not presented. Valenti et al. [11] compared the accuracy of their proposed adaptive CF, Madgwick’s CF, and extended Kalman filter (EKF) using the publicly-available Micro Aerial Vehicles (MAV) datasets. The results also show that the accuracy of EKF is better than that of Madgwick’s CF. In addition, the similar conclusions are drawn by Kottath et al. [12] too. Therefore, regarding estimation accuracy, KFs are considered as the better choices other than conventional CFs. Yuan et al. [14] proposed a quaternion-based unscented Kalman filter (UKF) for heading estimation using a tiny multi-sensor system. The system was fixed on waist of pedestrian and the quadrotor unmanned aerial vehicle (UAV) to test the heading estimation accuracy, and the results show that the mean heading estimation errors are less than 10° and 5° respectively. Ettlinger et al. [17] found that the systematic deviations in the observed data caused significant divergence between the estimated and the reference trajectory, and thus proposed a Gauss-Helmert model-based Kalman filter for reliability analysis in orientation determination with smartphone sensors. Deng et al. [4] proposed a quaternion-based EKF for heading estimation using smartphone-embedded sensors. According to their tests, location trace deviated from the ground truth significantly after a turn of 180 degrees without the assistance of WiFi localization. Li et al. [8] proposed a hybrid positioning algorithm based on EKF for the fusion of visible light information and the outputs of accelerometer and gyroscope. Visible light positioning results are used as the measurements to correct the states estimated by the PDR method. From their experiments, we find that the location trace produced by PDR diverges from the true path severely, but the result of hybrid algorithm is much better. Wang et al. [2] proposed to fuse PDR and floor map matching using an adaptive unscented Kalman filter (AUKF) algorithm. Experiments in an office building indicate that the location trace of PDR diverges in a short time period, but with the correction of the floor map, the location trace almost overlaps with the true path.

The state-of-the-art of the MEMS sensors-based indoor location tracking approaches reveals that PDR based on conventional filters with constant noise levels are easily affected by dynamic conditions, and other localization techniques or external data are effective to improve the filtering performance. Actually, besides the above presented solutions, location accuracy of conventional filters can also be improved by using adaptive methods. For example, Ding et al. [18] proposed a process noise scaling algorithm for autonomously tuning the process noise covariance to the optimal magnitude. Hu et al. [19] investigated two adaptive algorithms which were based on fading memory and variance component estimation respectively, and found that both algorithms perform better than conventional KF, and the variance component estimation filter achieves the best positioning accuracy. Li et al. [20] proposed an effective adaptive Kalman filter for attitude and heading estimation. When filtering, the noise variance matrix R is tuned by a three-segment function that is constructed depending on the level of acceleration. Zheng et al. [21] proposed a robust adaptive UKF with a two-step adaptive scheme. First, an innovation-based statistic is used to identify model errors, and then if model errors exist, two adaptive factors are applied to control the noise covariance matrices Q and R by balancing the last noise covariance matrices and the estimated ones. In summary, there are three kinds of adaptive filtering algorithms [18,22], such as the covariance scaling-based adaptive filter [19,21,23,24,25,26,27,28], the multi-model adaptive estimation-based filter [29], and adaptive stochastic modelling-based filter [18,20,30,31]. Moreover, in order to control the influence of measurement outliers, Yang et al. [32] combined robust estimation and adaptive filtering and proposed the theory of adaptively robust Kalman filtering for kinematic navigation and positioning. Yang also summarized the models and the judging statistics for constructing adaptive factors systematically, and explained the relations between their proposed algorithm and other filters in detail. Although these adaptive filtering algorithms have been widely applied in various fields, the application in PDR has been rarely investigated.

Considering pedestrians’ complex walking patterns, it is challenging to track or position them with their smart devices (smart phone, smart watch, etc.). As a result, in order to improve the tracking performances of PDR, a method based on robust adaptive Kalman filtering (RAKF) is proposed for heading estimation. Outputs from gyroscope, accelerometer and magnetometer sensors are used. To resist the negative impacts from measurement outliers and state model disturbances, a maximum likelihood-type estimator (M-estimator)-based model is used in combination with an adaptive factor. Generally, the contributions of our work can be summarized as follows:

- A heading estimation approach based on RAKF is proposed for PDR. Compared with the conventional KF-based approach, the proposed one uses an M-estimator-based model to control measurement outliers, and employs a state discrepancy statistic-based adaptive factor to resist the negative impacts of state model disturbances.
- Static tests were conducted, and the results indicate the advantages of our proposed approach over the conventional KF-based approach are faster converging speed, and more accurate estimation. Dynamic tests were carried out, and results of PDR demonstrate that our proposed approach provides more accurate and robust estimates, compared with the conventional KF-based approach.
- It is found that the proposed approach handles the issue of sudden turn in pedestrian location tracking quite well, and alleviates the problem of error accumulation effectively.

In the rest of this paper, we first present the procedure for heading estimation by using smart phone-embedded MEMS sensors in Section 2. After that, we explain the proposed RAKF algorithm in detail in Section 3. In Section 4, we show the experiments and results. At last, we draw conclusions and future work in Section 5.

## 2. Heading Estimation for PDR Based on Smart Phone-Embedded MEMS Sensors

**Figure 1:**
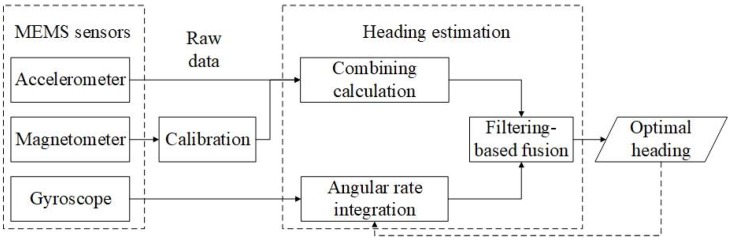
Architecture of the heading estimation approach.

Low cost MEMS sensors embedded in smart phones, such as accelerometers, magnetometers, and gyroscopes provide raw data for pedestrian speed estimation and heading estimation. In this paper, we assume that the heading of a pedestrian and that of his/her smart phone coincide. Thus, only the heading of the smart phone needs to be determined. As Figure 1 presents, raw data from the three sensors are used in two ways, the acceleration and magnetic field data are combined to calculate absolute headings, and the angular rate is used to integrate relative headings. The two kinds of headings are then fused using a filtering algorithm to obtain optimal values which may be used iteratively in the angular rate integration.

### 2.1. Heading Representation and Determination

Attitude and heading for a rigid body are always handled together. To represent the attitude heading, we define an orthogonal body frame (*X-Y-Z*) *B* in which *Y* and *Z* axes link up with the forward and up directions respectively, and *X* axis points to the right. Commonly, the attitude is determined by the rotation matrix with respect to the ENU (East (*X*)—North (*Y*)-Up (*Z*)) frame (also named navigation frame *N*). Define vector $X^{n}$ in frame *N*, and the corresponding vector $X^{b}$ in frame *B*, the mapping between the two vectors can be expressed as:

(1) $$X^{b}=C_{n}^{b}X^{n}$$

where, $C_{n}^{b}$ represents the rotation matrix from the frame *N* to the frame *B*. To be specific, suppose the frame *N* first rotate around *Z* axis with an angle *ψ*, and then rotate around *X* axis about an angle *θ*, and finally rotate around *Y* axis with an angle *φ*, the rotation matrix $C_{n}^{b}$ will be calculated as:

(2) $$C_{n}^{b}=\left[\begin{array}{ccc} \cos\varphi & 0 & -\sin\varphi \\ 0 & 1 & 0\\ \sin\varphi & 0 & \cos\varphi \end{array}\right]\left[\begin{array}{ccc} 1 & 0 & 0\\ 0 & \cos\theta & \sin\theta \\ 0 & -\sin\theta & \cos\theta \end{array}\right]\left[\begin{array}{ccc} \cos\psi & \sin\psi & 0\\ -\sin\psi & \cos\psi & 0\\ 0 & 0 & 1 \end{array}\right]$$

and it can be further written as:

(3) $$C_{n}^{b}=\left[\begin{array}{ccc} \cos\varphi \cos\psi -\sin\psi \sin\theta \sin\varphi & \cos\varphi \sin\psi +\cos\psi \sin\theta \sin\varphi & -\cos\theta \sin\varphi \\ -\sin\psi \cos\theta & \cos\theta \cos\psi & \sin\theta \\ \sin\varphi \cos\psi +\sin\psi \sin\theta \cos\varphi & \sin\varphi \sin\psi -\cos\psi \sin\theta \cos\varphi & \cos\theta \cos\varphi \end{array}\right]$$

According to the definition of body frame, *ψ*, *θ*, and *φ* are called heading, pitch, and roll angles respectively. It is noticed that $C_{n}^{b}$ will be different with respect to other rotational orders [33].

Since Euler angles have the problems of singularity and lower computation efficiency [14], quaternion is designed to replace it for attitude representation. A quaternion **q** is a 4-tuple:

(4) $$q={\left[\begin{array}{cc} q_{0} & e \end{array}\right]}^{T}$$

where $q_{0}$ is the scalar part, and $e={\left[\begin{array}{ccc} q_{1} & q_{2} & q_{3} \end{array}\right]}^{T}$ denotes the vector part. In this paper, unit quaternion that is with the constraint of unity norm:

(5) $$q_{0}^{2}+q_{1}^{2}+q_{2}^{2}+q_{3}^{2}=1$$

is used. Likewise, a unit quaternion can also be used to represent the attitude of a rigid body. Consider the vectors defined above, the mapping can be expressed as [34,35]:

(6) $$\left[\begin{array}{c} 0\\ X^{b} \end{array}\right]=q⊗\left[\begin{array}{c} 0\\ X^{n} \end{array}\right]⊗q^{-1}$$

where ⊗ indicates the quaternion multiplication, **q**^−1^ is the inverse of the quaternion **q**:

(7) $$q^{-1}={\left[\begin{array}{cc} q_{0} & -e \end{array}\right]}^{T}$$

According to the matrix form of quaternion multiplication [34], (6) can be expanded as:

(8) $$\left[\begin{array}{c} 0\\ X^{b} \end{array}\right]=\bar{M}{\left(q\right)}^{T}M\left(q\right)\left[\begin{array}{c} 0\\ X^{n} \end{array}\right]$$

where M(q) is the quaternion matrix function [34], and $\bar{M}\left(q\right)$ is its conjugate form. At last, we can get:

(9) $$\left[\begin{array}{c} 0\\ X^{b} \end{array}\right]=\left[\begin{array}{cc} 1 & 0^{T}\\ 0 & C_{n}^{b}\left(q\right) \end{array}\right]\left[\begin{array}{c} 0\\ X^{n} \end{array}\right]$$

A similar equation as (1) can be derived from (9):

(10) $$X^{b}=C_{n}^{b}\left(q\right)X^{n}$$

where $C_{n}^{b}\left(q\right)$ is the rotation matrix formed by using quaternion:

(11) $$C_{n}^{b}\left(q\right)=\left[\begin{array}{ccc} q_{0}^{2}+q_{1}^{2}-q_{2}^{2}-q_{3}^{2} & 2\left(q_{1}q_{2}+q_{0}q_{3}\right) & 2\left(q_{1}q_{3}-q_{0}q_{2}\right)\\ 2\left(q_{1}q_{2}-q_{0}q_{3}\right) & q_{0}^{2}-q_{1}^{2}+q_{2}^{2}-q_{3}^{2} & 2\left(q_{2}q_{3}+q_{0}q_{1}\right)\\ 2\left(q_{1}q_{3}+q_{0}q_{2}\right) & 2\left(q_{2}q_{3}-q_{0}q_{1}\right) & q_{0}^{2}-q_{1}^{2}-q_{2}^{2}+q_{3}^{2} \end{array}\right]$$

Inspection of (3) and (11) yields the calculation of the attitude heading:

(12) $$\theta =\arcsin\left(2\left(q_{2}q_{3}+q_{0}q_{1}\right)\right)$$

(13) $$\varphi =\arctan\left(\frac{2\left(q_{1}q_{3}-q_{0}q_{2}\right)}{q_{0}^{2}-q_{1}^{2}-q_{2}^{2}+q_{3}^{2}}\right)$$

(14) $$\psi =\arctan\left(\frac{2\left(q_{1}q_{2}-q_{0}q_{3}\right)}{q_{0}^{2}-q_{1}^{2}+q_{2}^{2}-q_{3}^{2}}\right)$$

### 2.2. Heading Estimation Using Acceleration and Magnetic Field

Having defined how to represent headings, accelerations and magnetic fields can be used to estimate meaningful headings for PDR.

#### 2.2.1. Magnetometer Calibration

**Figure 2:**
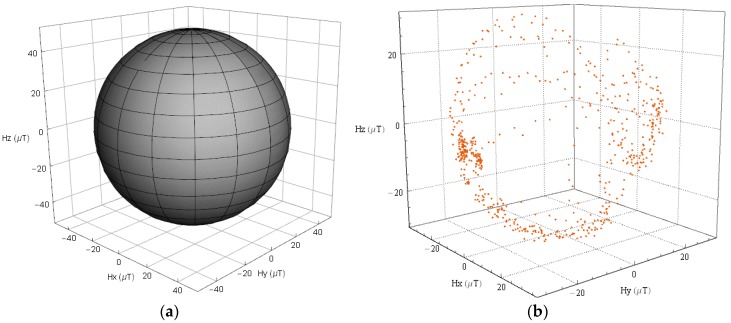
3-dimension locus magnetic field measurements, (**a**) error-free, (**b**) contaminated by hard iron, and scale factor error.

Magnetometers are essential for estimating absolute orientation, however, they often lack calibration, so that the outputs are easily contaminated by hard iron, soft iron, and scale factor errors. These errors can bias the magnetometer outputs, or be superimposed on the outputs. Methods for removing the negative impacts caused by these errors are needed. In this paper, we assume that the outputs of the magnetometer in a smart device are mainly corrupted by the hard iron and scale factor errors. Thus, a method that recovers the locus of error-free magnetic field measurements as Figure 2a presents from an altered locus as Figure 2b presents is applied. 

In general, the procedure can be summarized as follows:

(1) Constructing an ellipsoid model

We can see from Figure 2b that the magnetic field measurements at a given geographical location without calibration approximates an ellipsoid, thus an ellipsoid model that can adjust the bias and non-uniform scale is constructed:

(15) $$R^{2}={\left(\frac{m_{x}-\Delta m_{x0}}{1+sf_{x}}\right)}^{2}+{\left(\frac{m_{y}-\Delta m_{y0}}{1+sf_{y}}\right)}^{2}+{\left(\frac{m_{z}-\Delta m_{z0}}{1+sf_{z}}\right)}^{2}$$

where *m_x_*, *m_y_*, and *m_z_* denote the raw magnetometer measurements of a device in its body frame, *sf_x_*, *sf_y_*, and *sf_z_* denote the scale factors, $\Delta m_{x0}$, $\Delta m_{y0}$, and $\Delta m_{z0}$ denote the hard iron-caused biases, *R* denotes the ellipsoid radius.

(2) Estimating the parameters of the model

To fit the best ellipsoid and to estimate the six parameters accurately, enough measurements that span the entire Euler angle space at a given location should be collected. With the collected data, a least square (LS) estimation algorithm can be used to approximate the model. Detailed implementation of the LS algorithm refers to [36].

(3) Correcting the magnetic field measurements

With the estimated two tuples of parameters, magnetometer outputs can be calibrated. Raw measurements **m** (*m_x_*, *m_y_*, *m_z_*) are first shifted according to a vector Δ**m** (Δ*m_x_*_0_, Δ*m_y_*_0_, Δ*m_z_*_0_). Then, the measurements are scaled depending on a vector **s** (1 + *sf_x_*, 1 + *sf_y_*, 1 + *sf_z_*). Finally, we will obtain calibrated measurements $\hat{m}$ (${\hat{m}}_{x}$, ${\hat{m}}_{y}$, ${\hat{m}}_{z}$).

(16) $$\hat{m}=C_{sf}\left(m+\Delta m\right)$$

where **C***_sf_* = **s** · **I**_3×3_ denotes a scale transformation matrix.

#### 2.2.2. Heading Calculation

Once the magnetometer is calibrated, absolute headings with better accuracy can be obtained. Quaternion heading **q***_am_* can be directly derived from acceleration vector and calibrated magnetic field vector $\hat{m}$ by solving the Wahba’s problem [37]. Valenti et al. [13] proposed to decompose **q***_am_* into two quaternions, **q***_a_* and **q***_m_* which are determined by accelerations and magnetic field, respectively:

(17) $$q_{am}=q_{a}⊗q_{m}$$

(18) $$q_{a}=\{\begin{array}{c} {\left[\begin{array}{cccc} \lambda_{1} & -\frac{a_{y}}{2\lambda_{1}} & \frac{a_{x}}{2\lambda_{1}} & 0 \end{array}\right]}^{T},a_{z}\geq 0\\ {\left[\begin{array}{cccc} -\frac{a_{y}}{2\lambda_{2}} & \lambda_{2} & 0 & \frac{a_{x}}{2\lambda_{2}} \end{array}\right]}^{T},a_{z}<0 \end{array}$$

where *a_x_*, *a_y_*, and *a_z_* denote the accelerometer measurements of a device in its body frame, $\lambda_{1}=\sqrt{\frac{a_{z}+1}{2}}$, and $\lambda_{2}=\sqrt{\frac{1-a_{z}}{2}}$. Then the calibrated magnetic field vector is rotated using the quaternion **q***_a_*:

(19) $$l=C_{n}^{b}\left(q_{a}\right)\cdot \hat{m}$$

where **l** is the rotated magnetic field vector. Then the quaternion **q***_m_* is futher derived from **l**:

(20) $$q_{m}=\{\begin{array}{c} {\left[\begin{array}{cccc} \frac{\sqrt{\Gamma +l_{x}\sqrt{\Gamma }}}{\sqrt{2\Gamma }} & 0 & 0 & \frac{l_{y}}{\sqrt{2}\sqrt{\Gamma +l_{x}\sqrt{\Gamma }}} \end{array}\right]}^{T},l_{x}\geq 0\\ {\left[\begin{array}{cccc} \frac{l_{y}}{\sqrt{2}\sqrt{\Gamma -l_{x}\sqrt{\Gamma }}} & 0 & 0 & \frac{\sqrt{\Gamma -l_{x}\sqrt{\Gamma }}}{\sqrt{2\Gamma }} \end{array}\right]}^{T},l_{x}<0 \end{array}$$

where *l_x_* and *l_y_* denote *X* and *Y* components of **l**, $\Gamma =l_{x}^{2}+l_{y}^{2}$.

### 2.3. Heading Estimation Using Angular Rate

The angular rates output by the gyroscope can also be used to estimate quaternion attitude headings which represent the changed value relative to the initial quaternion, and the estimation is based on a differential equation:

(21) $$\frac{dq}{dt}=\frac{1}{2}q⊗s_{\omega }$$

where $s_{\omega }$ is constructed with the gyroscope output:

(22) $$s_{\omega }={\left[\begin{array}{cccc} 0 & \omega_{x} & \omega_{y} & \omega_{z} \end{array}\right]}^{T}$$

where *ω_x_*, *ω_y_*, and *ω_z_* are the *X*, *Y*, and *Z* components of the gyroscope output in a device’s body frame. In order to obtain the results at different time instants, the discrete form of (21) should be used:

(23) $$q_{\omega ,t}=q_{\omega ,t-1}+\left(\frac{1}{2}q_{\omega ,t-1}⊗s_{\omega ,t}\right)\Delta t$$

Using the quaternion matrix function, (23) can be further expanded as:

(24) $$q_{\omega ,t}=\left[I_{4\times 4}+\frac{\Delta t}{2}M\left(s_{\omega ,t}\right)\right]q_{\omega ,t-1}$$

where $M\left(s_{\omega ,t}\right)$ is the quaternion matrix function of $s_{\omega ,t}$.

The above two methods for heading estimation can be combined to produce more robust and accurate results, and a frame of KF is applied in this paper. In the following, the process of RAKF is explained in detail.

## 3. Robust Adaptive Kalman Filtering for Heading Estimation

### 3.1. State and Measuring Models for Heading Estimation

According to the above equations for calculating quaternion headings, the state and measuring models are designed as follows:

Let $X_{k}$ represent the state at time *k*, and $F_{k}=I_{4\times 4}+\frac{\Delta t}{2}M\left(s_{\omega ,k}\right)$, the state model can be formed as:

(25) $$X_{k}=F_{k}X_{k-1}+w_{k}$$

where $w_{k}$ denotes the model noise.

Let **Z***_k_* denote the measurement at time *k*:

(26) $$Z_{k}=q_{am,k}$$

where $q_{am,k}$ is calculated using (17). To avoid the discontinuities of headings caused by (18) and (20), **Z***_k_* has to be changed by checking the difference between current predicted state $X_{k}$ and itself:

(27) $$\{\begin{array}{l} Z_{k}=Z_{k},\;d_{k}>0\\ Z_{k}=-Z_{k},d_{k}\leq 0 \end{array}$$

where $d_{k}=Z_{k}\cdot X_{k}$ is the dot product of two quaternions. A linear function is enough to construct the measuring model:

(28) $$Z_{k}=HX_{k}+v_{k}$$

where **H** is an identity matrix **I**_4×4_, and $v_{k}$ denotes the model noise.

Since a unit quaternion is used in our designed algorithm, the initial state value $X_{0}=q_{\omega ,0}$ and all the measurements $Z_{1:k}=\left\{Z_{i},i=1,\cdots ,k\right\}$ should be normalized before they are inputted into the process of filtering. Given the models for heading estimation are designed above, a RAKF algorithm is used for obtaining optimal results. The algorithm consists of two procedures, predicting and updating which are presented in the subsequent sections.

### 3.2. Predicting

- Computing the predicted state ${\hat{X}}_{k|k-1}$

According to the state model in (25), the predicted state can be calculated as:

(29) $${\hat{X}}_{k|k-1}=F_{k}{\hat{X}}_{k-1}$$

- Computing the predicted state error variance matrix ${\hat{P}}_{k|k-1}$:

(30) $${\hat{P}}_{k|k-1}=F_{k}{\hat{P}}_{k-1}F_{k}^{T}+Q_{k}$$

where **Q***_k_* is the state model noise covariance matrix.

### 3.3. Updating

- Computing the gain matrix $K_{k}$

In the RAKF, the computation of $K_{k}$ is different from conventional implementation in the KF. To control the outliers in the measurements, an M-estimator-based robust estimation of the equivalent weight matrix ${\bar{P}}_{k}$ of the measurements is used. Among several formatting methods, we choose Huber’s approach [32]. Then, the diagonal ${\bar{p}}_{k_{ii}}$ and non-diagonal ${\bar{p}}_{k_{ij}}$ elements of ${\bar{P}}_{k}$ are determined as follows:

(31) $${\bar{p}}_{k_{ii}}=\{\begin{array}{l} \frac{1}{\sigma_{ii}},\;\left|r_{k_{i}}^{\prime }\right|\leq c\\ \frac{c}{\left|r_{k_{i}}^{\prime }\right|}\cdot \frac{1}{\sigma_{ii}},\;\left|r_{k_{i}}^{\prime }\right|>c \end{array}$$

(32) $${\bar{p}}_{k_{ij}}=\{\begin{array}{l} \frac{1}{\sigma_{ij}},\;\left|r_{k_{i}}^{\prime }\right|\leq c\;and\;\left|r_{k_{j}}^{\prime }\right|\leq c\\ \frac{c}{\max\left\{\left|r_{k_{i}}^{\prime }\right|,\left|r_{k_{j}}^{\prime }\right|\right\}}\cdot \frac{1}{\sigma_{i,j}},\;\left|r_{k_{i}}^{\prime }\right|>c\;or\;\left|r_{k_{j}}^{\prime }\right|>c \end{array}$$

where $\sigma_{ii}$ and $\sigma_{ij}$ are diagonal and non-diagonal elements of the measurement noise covariance matrix **R***_k_*. *c* is a constant, and it is usually within the range of [1.3, 2.0]. $r_{k_{i}}^{\prime }$ denotes the standard residual, and it is calculated by:

(33) $$\left|r_{k_{i}}^{\prime }\right|=\left|\frac{r_{k_{i}}}{\sigma_{r_{k_{i}}}}\right|$$

where $r_{k_{i}}$ is the residual of the measurement $z_{k_{i}}$, and $\sigma_{r_{k_{i}}}$ is the mean deviation of $r_{k_{i}}$:

(34) $$r_{k_{i}}={\left(Z_{k}-{\hat{Z}}_{k|k-1}\right)}_{i}$$

where ${\hat{Z}}_{k|k-1}$ is the predicted measurement which is calculated depending on the measuring model in (28):

(35) $${\hat{Z}}_{k|k-1}=H{\hat{X}}_{k|k-1}$$

In order to control the influence of dynamic model error, an adaptive factor is applied for correcting the predicted state error variance matrix ${\hat{P}}_{k|k-1}$. Before calculating the adaptive factor, a kind of state discrepancy statistic for judging the state model errors [26,32] is chosen as:

(36) $$\Delta {\tilde{X}}_{k}=\frac{\left\|{\tilde{X}}_{k}-{\hat{X}}_{k|k-1}\right\|}{\sqrt{tr\left({\hat{P}}_{k|k-1}\right)}}$$

where *tr*(·) stands for the trace of a matrix. ${\tilde{X}}_{k}$ is a least-square estimator of the state:

(37) $${\tilde{X}}_{k}={\left(\phi_{k}^{T}P_{k}\phi_{k}\right)}^{-1}\phi_{k}^{T}P_{k}Z_{k}$$

where $P_{k}=R_{k}^{-1}$ denotes the weight matrix. To avoid measurement outliers, equivalent weight matrix ${\bar{P}}_{k}$ can be applied in (37).

With the chosen statistic $\Delta {\tilde{X}}_{k}$, a two-segment function is applied for calculating the adaptive factor:

(38) $$\partial_{k}=\{\begin{array}{l} 1,\;\Delta {\tilde{X}}_{k}\leq c0\\ \frac{c0}{\Delta {\tilde{X}}_{k}},\;\Delta {\tilde{X}}_{k}>c0 \end{array}$$

where *c*0 is a constant which can be tuned depending on practical implementation.

Having weaken the negative impacts from measurement outliers and state model errors, a proper gain matrix can be obtained:

(39) $$K_{k}=\frac{1}{\partial_{k}}{\hat{P}}_{k|k-1}H_{k}^{T}{\left(\frac{1}{\partial_{k}}H_{k}{\hat{P}}_{k|k-1}H_{k}^{T}+{\bar{P}}_{k}^{-1}\right)}^{-1}$$

- Computing the corrected state ${\hat{X}}_{k}$:

(40) $${\hat{X}}_{k}={\hat{X}}_{k|k-1}+K_{k}r_{k}$$

The state needs to be normalized further:

(41) $${\hat{X}}_{k}={\hat{X}}_{k}/\left\|{\hat{X}}_{k}\right\|$$

- Updating the state error variance matrix ${\hat{P}}_{k}$:

(42) $${\hat{P}}_{k}=\left(I-K_{k}H_{k}\right){\hat{P}}_{k|k-1}$$

Using the equations from (29) to (42), headings can be estimated iteratively in the frame of RAKF.

## 4. Experimental Evaluation

### 4.1. Experimental Setup

**Figure 3:**
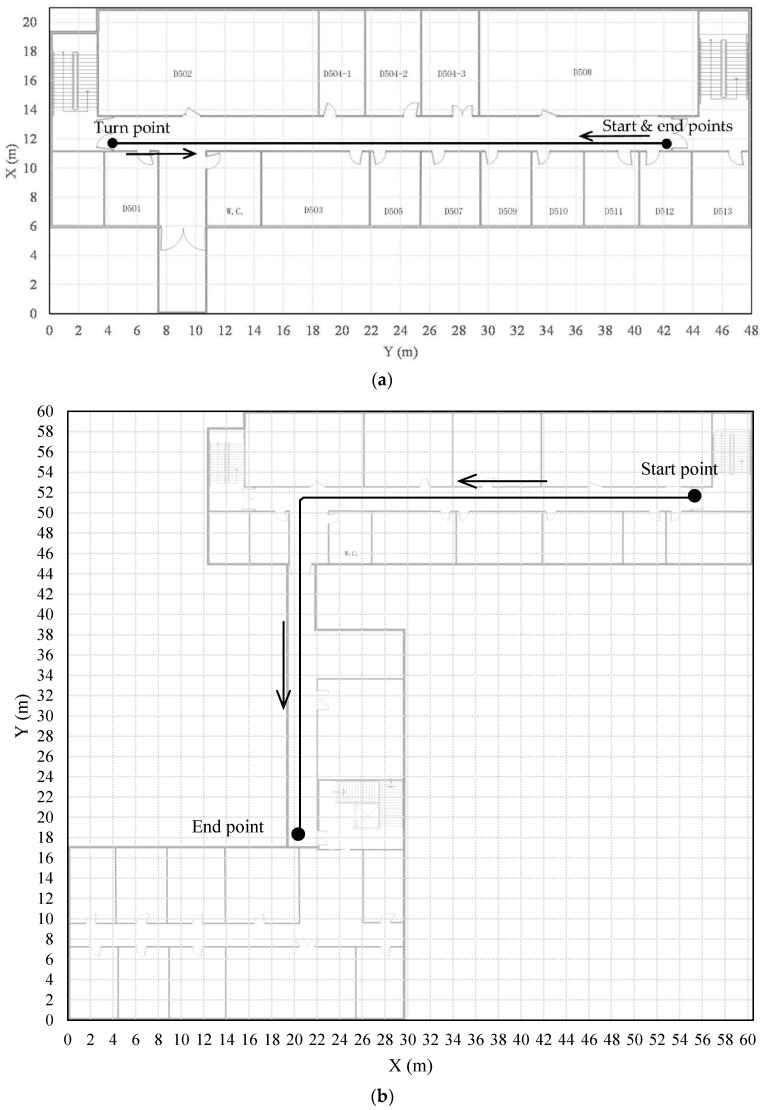
Floor plans of the sites for dynamic tests, (**a**) the first site, (**b**) the second site.

To evaluate the proposed heading estimation approach, we conducted extensive tests in two situations, static and dynamic. In the static tests, a Xiaomi 5 smart phone was put still on a table in an office, collect data covering more than ten minutes. All the heading results calculated from accelerations and magnetic field were averaged to obtain the reference heading value. Additionally, dynamic tests were performed in the corridors on the fifth and the seventh floors in the research building for the School of Geography and Planning at Sun Yat-sen University. The floor plans are presented in Figure 3a,b, respectively. We employed five persons to participate in collecting data. The participants had different heights, different weights, and different walking postures. Table 1 lists the detailed information of each participant. They were all asked to hold the Xiaomi 5 smart phone on their chest to collect data along labeled traces which are marked by black lines in Figure 3a,b respectively. For the tests in the first site on the fifth floor, three participants were involved in collecting data where they walked back and forth twice, and finally returned back to the start point after three sharp turns. The length of the location traces that they walked is as long as 150.4 m each. Whereas for the tests in the second site on the seventh floor, all of the five participants walked from the start point to the end point once. The length of each traces is about 68 m. A Xiaoyi 4kplus sports camera was used to record their walking, and relative accurate positions were derived from the videos to construct the reference traces.

Conventional KF is used as the baseline for comparisons, and the noise covariance matrix of both the measurements and the states are set differently in the static and dynamic tests. The matrices are empirically determined depending on the standard deviation of each measurement outputted by the smartphone. For static tests, **Q** = 10^−10^ * **I**_4×4_, **R** = 10^−6^ * **I**_4×4_, and for dynamic tests, **Q** = 10^−8^ * **I**_4×4_, **R** = 10^−6^ * **I**_4×4_. Moreover, the sampling frequency of data is 50 Hz.

### 4.2. Results and Analysis

#### 4.2.1. Performances on Heading Estimation in the Static Tests

**Figure 4:**
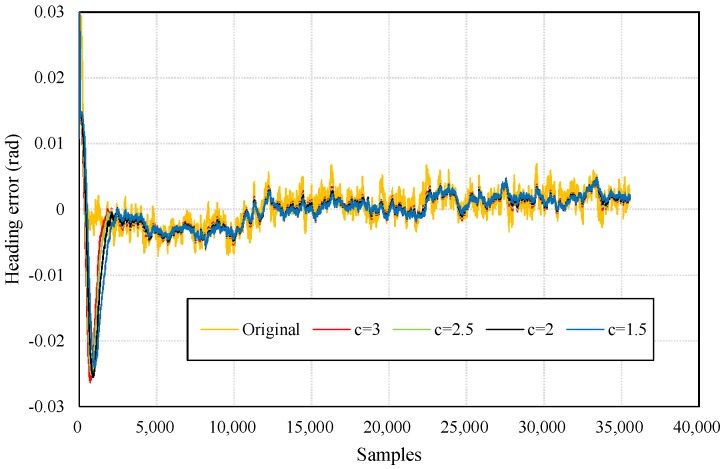
Heading errors with respect to different values of the robust parameter *c.*

Robust estimation can control the outputs of KF by using a parameter for determining which part of the measurements may cause negative influences. In this paper, the weights of the “negative measurements” are reduced to alleviate their impacts. However, if initial values for KF have not given properly, robust estimation can result slower convergence. Figure 4 presents heading errors of KF and its variants (Robust KF, RKF) with different values of the robust parameter. We find that RKFs produce significant heading errors at the beginning of filtering, but converge to relatively smooth results after a time period. Morever, the smaller the parameter is, the smoother the results are during the subsequent time period. Thus, the value in subsequent experiments is set to 1.5 which means the measurements with absolute errors over 1.5*σ* (mean squared error) will be handled.

**Figure 5:**
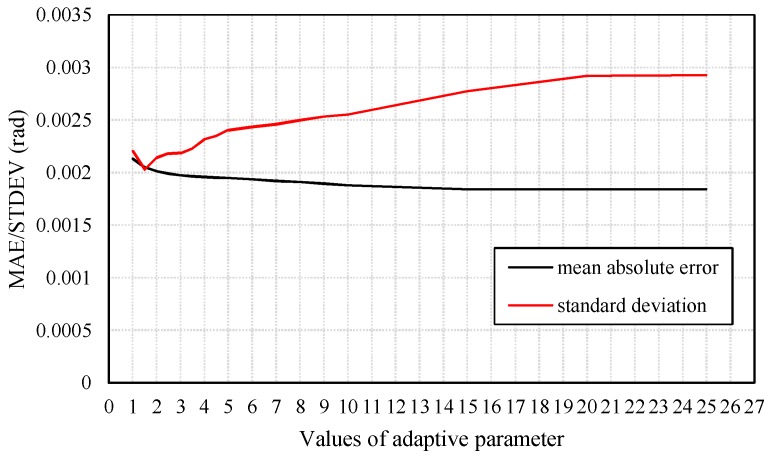
Standard deviations and mean values of absolute heading errors with respect to different values of the adaptive parameter *c*0.

**Figure 6:**
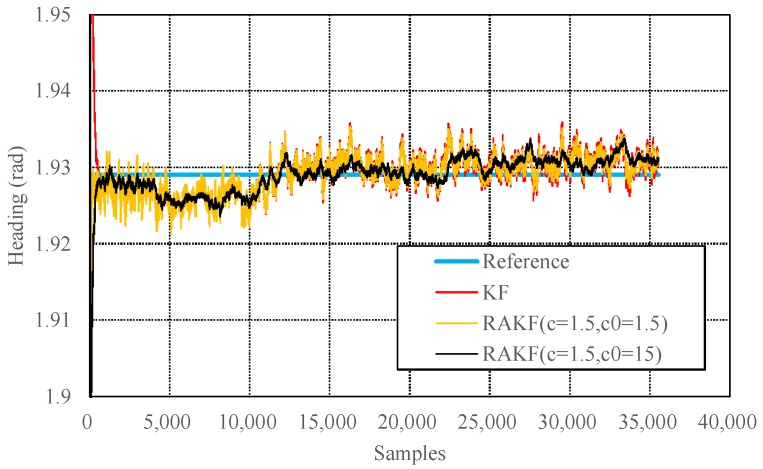
Comparisons of the performances on heading estimation of different algorithms in the static test.

The state discrepancy statistic-based adaptive method determines the adaptive factor depending on the difference between the predicted state and the result estimated using the acceleration and magnetic field data. The adaptive factor can directly change the accuracy and precision of the filtering results. Figure 5 presents mean values and standard deviations of absolute heading errors with respect to different values of adaptive parameters. The chosen of the adaptive parameter should balance the two aspects. Figure 6 presents results produced by KF and RAKFs with two different adaptive parameters. We can see that the results produced by the RAKF with an adaptive parameter of 1.5 converge faster but fluctuate with a larger amplitude, whereas the outputs of the RAKF with an adaptive parameter of 15 are smooth but converge slower. We adopt the average value of all the heading estimations from accelerometer and magnetometer as the true value to obtain the statistical results of estimation errors of KF and RAKF. Table 2 presents the results, and we can see that RAKFs estimate more accurate and steady headings than KF, the mean errors decrease about 8.2% and 17.6% respectively, and the standard deviations decrease about 38.5% and 15.8% respectively. Additionally, the results indicate special characteristics of RAKFs with different adaptive parameters.

#### 4.2.2. Performances on Heading Estimation in the Dynamic Tests

To further verify the superiority of the proposed RAKF in heading estimation, dynamic tests were conducted at two test sites. Since reference headings in dynamic tests are hard to obtain, estimation errors cannot be presented straightforwardly. Fortunately, location tracking performance of PDR can reflect heading estimation performance to some extent. Therefore, in order to examine heading errors, headings estimated by KF and RAKF are applied respectively in PDR location tracking which is based on conventional EKF.

An EKF-based PDR always consists of three parts, heading estimation, speed estimation, and location tracking. Except heading estimation, the other two parts are explained simply in the following. Detailed implementation refers to [38]. Speed estimation contains two steps, stride detection and step length estimation. For stride detection, peaks of measured total acceleration are counted. For step length estimation, a one-parameter nonlinear model [7,38] is employed:

(43) $$StepLength\approx \sqrt[{4}]{A_{\max}-A_{\min}}\times K$$

where $A_{\max}$ (or $A_{\min}$) is the maximum (or minimum) vertical acceleration in a single step and *K* is a constant. An assumption is that the leg is a lever of fixed length while the foot is on the ground. Location tracking is based on the primary theory of dead reckoning [7,38], and it is implemented in the frame of EKF, as presented in [38].

Depending on different walking patterns of participants, *K* in (43) is set separately. The value of *K* for each participant is presented in Table 1. Other settings for the filtering, such as model noise variances, robust parameter and adaptive parameter are with the same values in which the robust and the adaptive parameters are set as 1.5 and 3, respectively.

- Results of the tests in the first site

**Figure 7:**
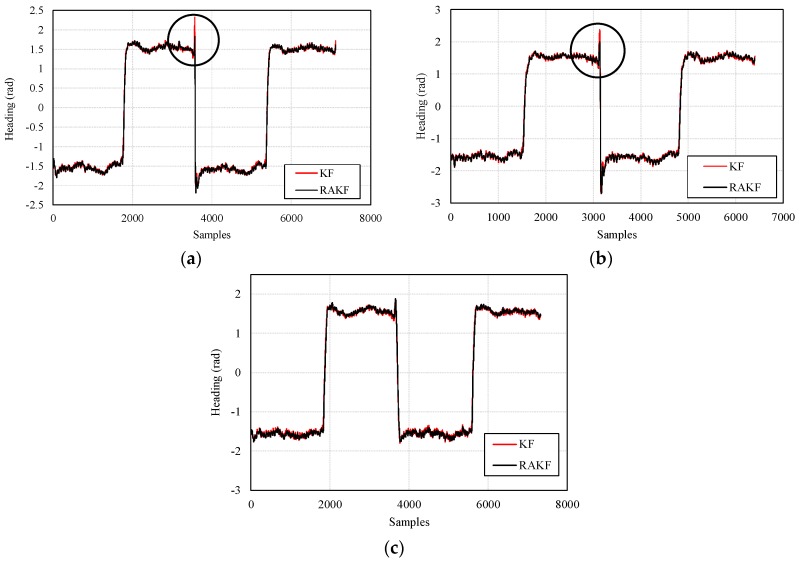
Comparisons of the performances on heading estimation of KF and RAKF with respect to three participants, (**a**) participant 1, (**b**) participant 2, and (**c**) participant 3.

The heading estimation results of KF and RAKF are presented in Figure 7. We can see that, the results of RAKF are smoother than those of KF. Noticeably, the most important improvement that is marked by black circles in Figure 7a,b of RAKF for heading estimation is the ability of controlling outliers, as well as the adaptation to sudden heading changes, compared with KF. However, similar improvements cannot be found in Figure 7c which means sudden turns did not cause significant heading errors during participant 3’s walking. The results also indicate that different walking characteristics of the three participants bring about great challenges for heading estimation based on RAKF with constant robust and adaptive parameters.

**Figure 8:**
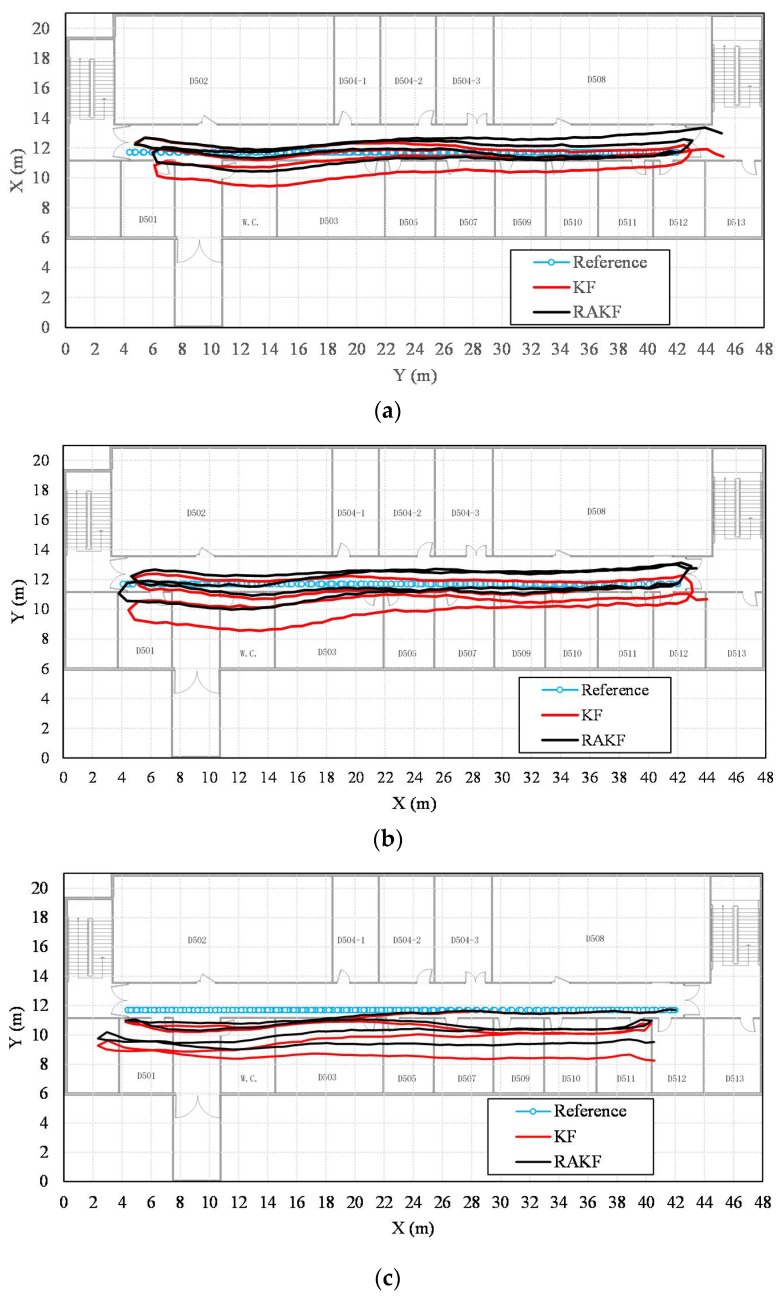
Comparisons of the performances on location tracking of KF and RAKF with respect to three participants, (**a**) participant 1, (**b**) participant 2, and (**c**) participant 3.

**Figure 9:**
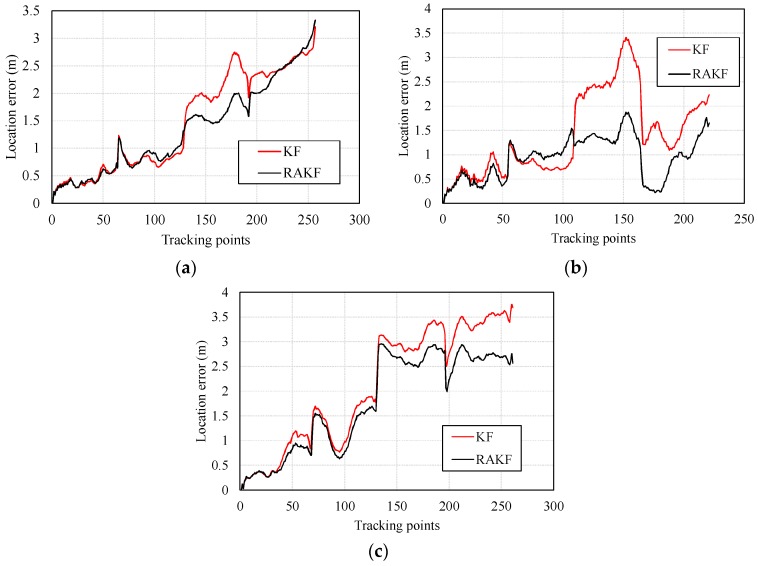
Distributions of location errors in location tracking of KF and RAKF with respect to three participants, (**a**) participant 1, (**b**) participant 2, and (**c**) participant 3.

More intuitive improvements can be observed in location tracking, performances of which are presented in Figure 8. For all three participants, the RAKF results approximate the reference trace better, compared with the results of KF. Location errors in tracking are further shown in Figure 9. All three figures indicate that KF and RAKF both provide low location errors at the beginning of tracking, but KF performs as worse as the walking distance becomes longer. The performances demonstrate that PDR suffers from location error accumulation, but RAKF is able to handle the problem to some extent. Finally, Table 3 gives the statistical results of location errors. Compared with KF, the outputs of RAKF are with higher accuracy and precision. The mean errors of RAKF’ outputs decrease 8.8%, 39.7%, 15.2% respectively, and the standard deviations of location errors decrease 10%, 53.2%,17.5%, respectively, compared with that of KF.

- Results of the tests in the second site

**Figure 10:**
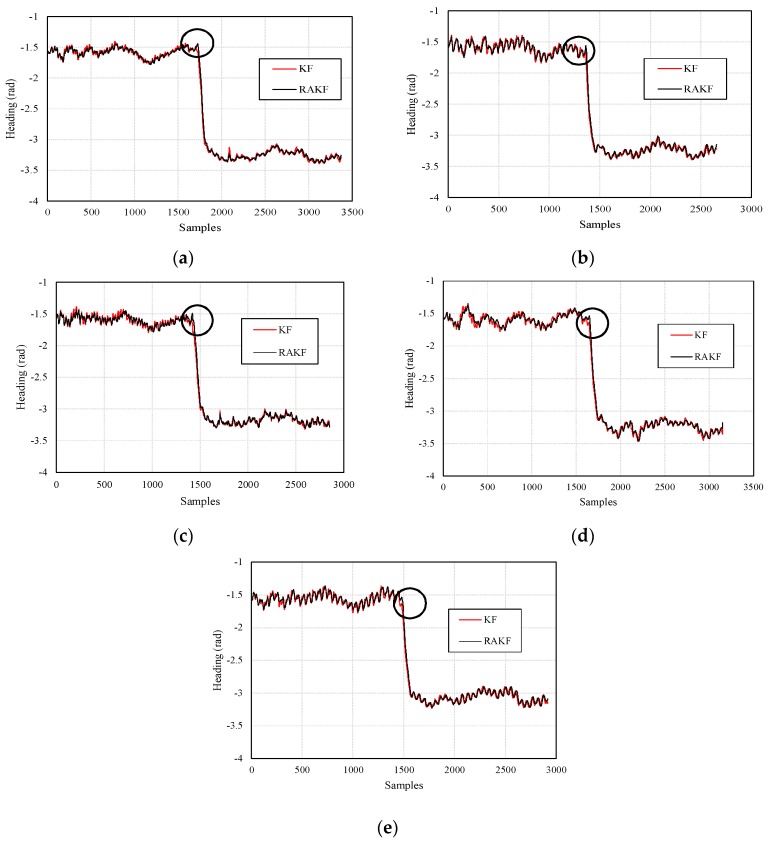
Comparisons of the performances on heading estimation of KF and RAKF regarding five participants, (**a**) participant 1, (**b**) participant 2, (**c**) participant 3, (**d**) participant 4, and (**e**) participant 5.

**Figure 11:**
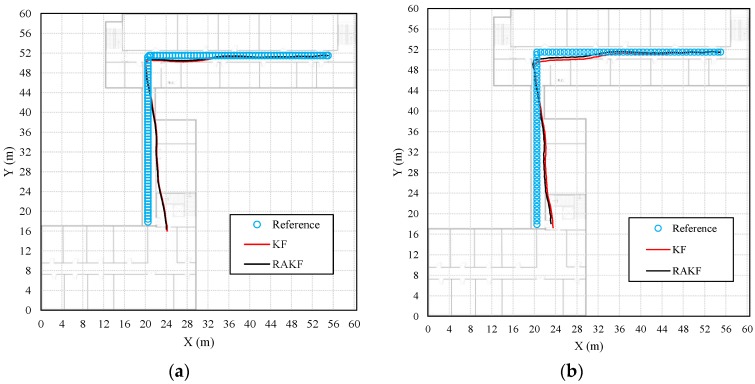

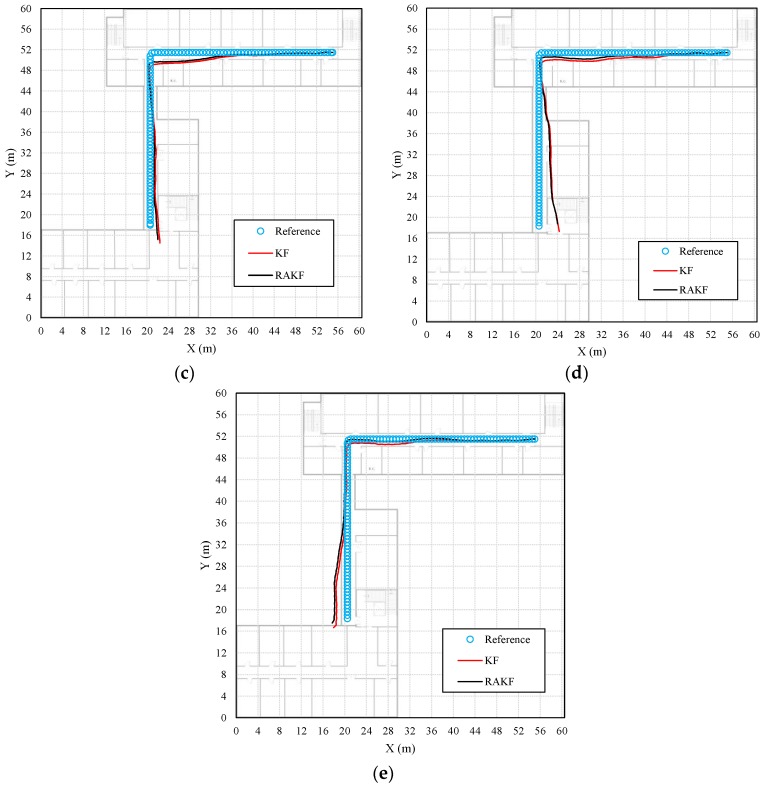
Comparisons of the performances on location tracking of KF and RAKF regarding five participants, (**a**) participant 1, (**b**) participant 2, (**c**) participant 3, (**d**) participant 4, and (**e**) participant 5.

**Figure 12:**
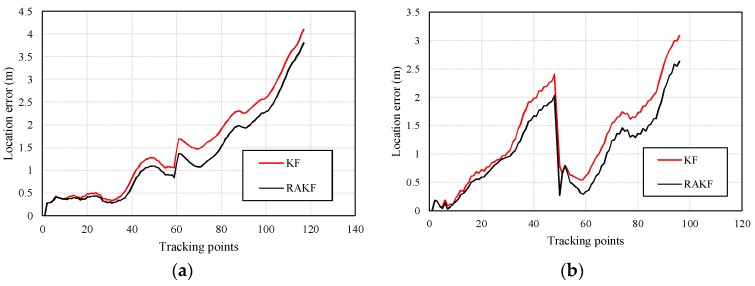

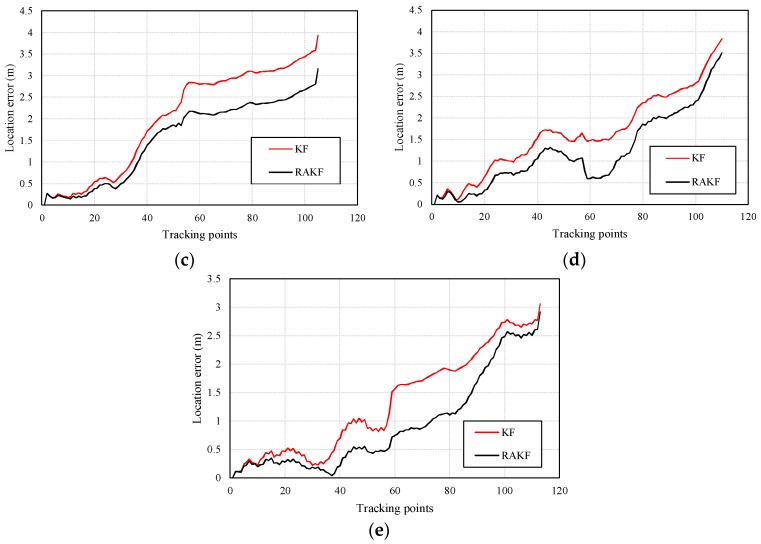
Distributions of location errors in location tracking of KF and RAKF regarding five participants, (**a**) participant 1, (**b**) participant 2, (**c**) participant 3, (**d**) participant 4, and (**e**) participant 5.

**Figure 13:**
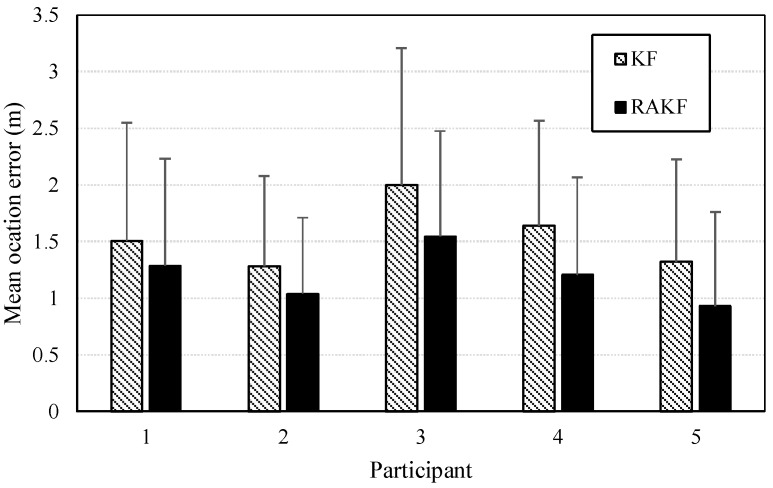
Mean values of location errors in location tracking of KF and RAKF regarding five participants (the error bar stands for the STD. = of location errors).

Similar heading estimation performances were obtained at the second site. The heading estimation results of KF and RAKF are presented in Figure 10. We still can see that the results of RAKF are smoother than those of KF. The adaptation of RAKF to sudden turns which are marked by black circles in Figure 10a–e is proven again. More intuitive performances can be observed in location tracking, results presented in Figure 11. For all five participants, the RAKF results approximate the reference trace better, compared with the results of KF. The changing of location errors in tracking are further drawn in Figure 12. The performances demonstrate that PDR suffers from location error accumulation, but RAKF is able to handle the problem to some extent. Finally, Figure 13 gives the mean and STD. errors of location tracking for five participants. Compared with KF, the outputs of RAKF are with higher accuracy and precision. The mean errors of RAKF’ outputs decrease 14%, 18%, 22%, 26%, and 29%, respectively, and the standard deviations of location errors decrease 9%, 15%, 23%, 7%, and 7.8%, respectively, compared with that of KF.

**Figure 14:**
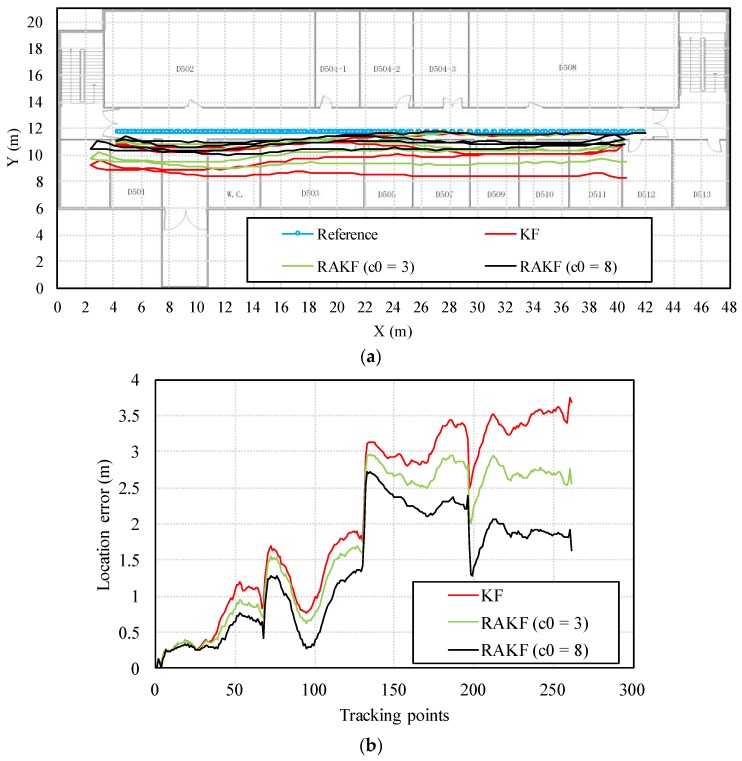
Location tracking performances in terms of (**a**) trajectory, (**b**) location error distribution of different algorithms regarding the participant 3.

We can find from the location tracking results of both test sites that the accuracy levels are different with the same setting of noise covariance matrices. Precisely, the noise covariance matrices used in Kalman filtering should be set specially for each pedestrian. However, this is impractical for wide implementation. The RAKF can alleviate the negative influence of imprecise setting of noise covariance matrices to some extent, but the best location accuracy performance is hard to obtain with constant robust and adaptive parameters. Taking location tracking of participant 3 in the first test site as an example, although the location accuracy provided by the proposed RAKF with the parameters *c* = 1.5, and *c*0 = 3 is higher than that of KF, RAKF with other setting of parameters can achieve even better performances. Figure 14 presents comparions on location tracking trajectory and location error distribution using different algorithms. We can find that RAKF with the parameters *c* = 1.5, and *c*0 = 8 performs much better than it with the parameters *c* = 1.5, and *c*0 = 3. Therefore, in our opinion, an automatic solution for determining the most suitable adaptive and further robust parameters are needed.

Generally, the above results demonstrate that KF can provide optimal heading estimations with proper models and the corresponding noise properties. However, for PDR, the statistical properties of pedestrians’ movements are changing dynamically and constant noise levels can result in divergent performances both in heading estimation and location tracking. Moreover, measurement outliers also corrupt the performance of PDR. The proposed RAKF can adapt to dynamic conditions, such as sudden turns during pedestrian’s walking, and it is robust in that constant parameters are effective for different persons. Moreover, the results also indicate that heading errors are some of the main error sources for location estimation using the PDR approach. It is necessary to obtain accurate headings, whether the PDR method is assisted by external techniques or not. 

Finally, we analyzed the computational time of the proposed approach. The KF and RAKF algorithms are implemented using C#, and the corresponding software runs on an 2.7 Hz Intel Core i5 processor. The average runtimes of one iteration of the filtering algorithms are listed in Table 4. The results demonstrate that our proposed RAKF is slightly slower than the KF. Nonetheless, the proposed RAKF improves the accuracy of heading estimation effectively.

## 5. Conclusions and Future Work

In this paper, the outputs of smart phone-embedded MEMS sensors, such as accelerometers, magnetometers, and gyroscopes are fused using RAKF for pedestrian heading estimation. To alleviate the negative influence of measurement outliers, an M-estimator-based model is applied for identifying and controlling them. Moreover, a state discrepancy statistic-based adaptive factor is used to reduce the effect of state model disturbances. Experiments under static and dynamic conditions are conducted to verify the superiority of the application of RAKF over KF. In the static tests, RAKF provides faster convergence speed and better accuracy, compared with KF. In the dynamic tests, the headings produced by RAKF and KF are input into a PDR method, respectively. Location tracking performances reveal that the headings estimated by RAKF lead to more accurate location estimations, especially in the situation of sudden turns during pedestrians’ walking. The results also tell us that it is necessary to estimate headings accurately, although there are other data or techniques, such as indoor graphs, WiFi positioning for enhancing the performance of PDR.

For PDR, each pedestrian may have special walking characteristics, which can result in a dedicated noise covariance matrix. Thus, the determination of adaptive parameters seems a cumbersome task which needs an automatic process. In the future, we will focus on studying an adaptive solution with the ability to automatically determine parameters. Moreover, tests about different carrying modes of the smart phone will be carried out, and the associating modifications to the filtering will be made.

## Figures and Tables

**Table 1:** Detailed information of all participants (*K* is the step length parameter used in Section 4.2.2).

Participant	Sex	Height (m)	Weight (Kg)	*K*
1	Male	1.66	59	0.36
2	Male	1.75	75	0.43
3	Male	1.71	60	0.39
4	Female	1.61	52	0.4
5	Male	1.64	65	0.37

**Table 2:** Statistical results of estimation errors of EKF and RAEKF.

Algorithms	Mean (Rad)	STDEV. (Rad)
KF	0.002232	0.003297
RAKF (*c* = 1.5, *c*0 = 1.5)	0.002049	0.002028
RAKF (*c* = 1.5, *c*0 = 15)	0.00184	0.002776

**Table 3:** Statistical results of location errors.

Participants	Error Metrics	KF	RAKF
Participant 1	Mean error (m)	1.48	1.35
	STD. error (m)	0.90	0.81
Participant 2	Mean error (m)	1.41	0.85
	STD. error (m)	0.94	0.44
Participant 3	Mean error (m)	2.10	1.78
	STD. error (m)	1.20	0.99

**Table 4:** Computational time of the heading estimation algorithms.

Algorithm	Average Time (ms)
KF	0.036610526
RAKF	0.040133333
